# GhmiR156-*GhSPL2* Module Regulates Anthocyanin Biosynthesis of Ray Florets in *Gerbera hybrida*

**DOI:** 10.3390/ijms27010318

**Published:** 2025-12-27

**Authors:** Mengdi Li, Bingbing Liao, Shuyuan Shi, Qishan Luo, Yanbo Chen, Xiaojing Wang, Yaqin Wang

**Affiliations:** 1 Guangdong Provincial Key Laboratory of Biotechnology for Plant Development, School of Life Sciences, South China Normal University, Guangzhou 510631, China; 2 Guangdong Provincial Key Laboratory of Utilization and Conservation of Food and Medicinal Resources in Northern Region, School of Biology and Agriculture, Shaoguan University, Shaoguan 512000, China

**Keywords:** *Gerbera hybrida*, anthocyanin biosynthesis, GhmiR156, *GhSPL2*

## Abstract

Anthocyanins, biosynthesized through the flavonoid pathway, critically determine floral coloration and ornamental value in plants. While floral development has been extensively studied in *Gerbera hybrida*, the microRNA-mediated regulation of anthocyanin biosynthesis remains unclear. In this study, we identified and characterized the precursor of gerbera microRNA156 (GhmiR156), which exhibits a typical stem-loop secondary structure. The mature GhmiR156 sequence shows 93.65% similarity with miR156 from other plants. Through target prediction analysis, we identified five potential target genes of GhmiR156, all encoding SQUAMOSA Promoter-Binding Protein-Like (SPL) transcription factors. Among these, the gene c35442.graph_c0, which shares the highest similarity with *AtSPL2* in Arabidopsis, was designated as *GhSPL2*. Expression analysis revealed an inverse correlation between GhmiR156 and *GhSPL2* across different tissues and developmental stages of ray florets. This regulatory relationship was further validated by RLM-5′RACE, which showed that GhmiR156 directly targets and cleaves *GhSPL2* mRNA, subsequently supported by dual-luciferase reporter assays and Western blotting analysis. Subcellular localization analysis indicated that GhSPL2 is a nuclear-localized protein, consistent with AtSPL2. Functional analyses revealed that overexpression of *GhSPL2* suppressed anthocyanin accumulation by downregulating key biosynthetic genes *GhPAL*, *GhF3H* and *GhUFGT*. Conversely, overexpression of GhmiR156 represses *GhSPL2* expression, thereby alleviating its inhibitory effect on anthocyanin accumulation in ray florets, and exhibits an increase in anthocyanin content. Collectively, our findings demonstrate that GhmiR156 fine-tunes the anthocyanin biosynthetic pathway through its target gene *GhSPL2*. This study provides new insights into the complex regulatory network governing anthocyanin biosynthesis in ornamental plants.

## 1. Introduction

Petals, as a central component of floral organs, serve as critical indicators for assessing the ornamental and economic value of plants. The morphological traits of flowers, including color and shape, play vital roles in protecting reproductive organs, attracting pollinators, and facilitating plant reproduction [1,2,3]. Among the factors influencing flower color, flavonoids, carotenoids and chlorophylls are recognized as the primary chemical pigments responsible for petal coloration [4,5,6,7]. Notably, anthocyanins, a class of flavonoids, are the major contributors to petal color [8,9,10]. The anthocyanin biosynthetic pathway involves two key groups of genes [11,12,13]. The first group comprises structural genes, such as chalcone synthase (*CHS*), chalcone isomerase (*CHI*), and flavanone-3-hydroxylase (*F3H*), which encode enzymes essential for anthocyanin production. The second group consists of regulatory factors, including MYB, basic helix-loop-helix (bHLH), and WD40 transcription factors; these proteins form the MYB-bHLH-WDR (MBW) complex to modulate the expression of structural genes. In addition, post-transcriptional regulation mediated by microRNAs (miRNAs) have been shown to significantly influence anthocyanin biosynthesis [14,15,16]. In *Arabidopsis thaliana*, miR156 targets *AtSPL9*, while miR828 targets *MYB75*, *MYB82*, *MYB90* and *MYB113*, both playing roles in anthocyanin regulation [17,18]. Similarly, miR399d in *Malus hybrid* ‘Royalty’ has been implicated in anthocyanin accumulation under phosphorus-deficient conditions [19]. Furthermore, miR172b in purple potato (*Solanum tuberosum* L.) has been linked to the regulation of anthocyanin types through its target gene, an *AP2*-like factor [20].

miRNAs are endogenous non-coding RNAs, typically 20–24 nucleotides in length, that regulate gene expression by cleaving target mRNAs or inhibiting their translation [21]. miRNAs play pivotal roles in various aspects of plant development, including leaf growth, floral transition and floral organ formation [13,20,22,23,24]. Among the most conserved miRNAs, miR156 has been extensively studied for its role in plant development [13,25,26,27], particularly through its targeting of SPL transcription factors [28,29,30]. The miR156-*SPL* module has been shown to regulate diverse processes, including flowering time, plant morphology, and anthocyanin biosynthesis [31,32,33]. While the miR156-*SPL* module has been well-characterized in Arabidopsis and fruit crops, only a few flower plants have a potential link between miR156-*SPL* and anthocyanin biosynthesis [30,34,35]. However, the specific mechanisms underlying this regulation vary across species, as evidenced by differences in miR156-*SPL* interactions in yellow-flowered tree peony (*Paeonia suffruticosa* Andr.) and herbaceous peony (*Paeonia lactiflora* Pall.) [36,37]. Therefore, these findings highlight the need for further investigation into the miR156-*SPL* regulatory network in ornamental plants.

*Gerbera hybrida*, a perennial herbaceous plant in the Asteraceae family, is known for its diverse flower colors, varied forms, long blooming period, and extensive range of cultivars. The inflorescence of gerbera, a capitulum, is primarily composed of three morphologically distinct types of florets arranged from the periphery to the center: ray florets, trans florets, and disk florets. The ray florets, located at the outermost region of the capitulum, exhibit a rich diversity in color—including red, yellow, orange, pink, white, and more. And, according to the length and coloring of ray florets, the developmental stages of inflorescence were divided into six distinct stages (S1–S6) [38]. Previous studies mainly focused on the regulation of transcription factors on the elongation and coloring of ray florets in gerbera [39,40,41,42,43,44,45], while the molecular mechanisms underlying miRNA-mediated regulation of ray floret coloration remain poorly understood.

In this study, we cloned and characterized GhmiR156 and its target gene *GhSPL2* in gerbera. Through bioinformatics analysis, RLM-5′RACE, dual-luciferase assay and Western blotting, we demonstrated that GhmiR156 targets and cleaves *GhSPL2* mRNA, thereby suppressing its expression. Functional studies revealed that GhmiR156 alleviates the inhibitory effect of *GhSPL2* on anthocyanin accumulation in ray florets. Collectively, our findings provide the first functional evidence for the miR156-*GhSPL2* regulatory module in gerbera, offering new insights into its role in anthocyanin biosynthesis and contributing to a deeper understanding of the regulatory network governing petal coloration in gerbera.

## 2. Results

### 2.1. Identification and Characterization of miR156 in Gerbera

**Figure 1:**
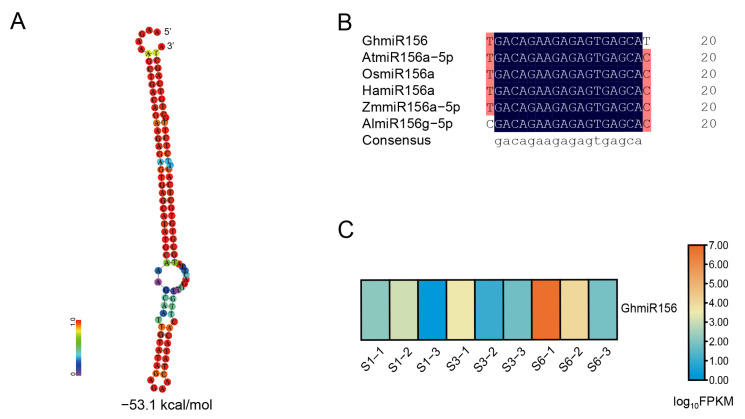
The identification of miR156 in gerbera. (**A**) Secondary structure of GhmiR156 precursor. The stem-loop structures are colored by base-pairing probabilities, red: high probability, purple: low probability. kcal/mol: the minimum free energy of the stem-loop structures. (**B**) Homology comparison of miR156s in gerbera and other plants. Gh, *Gerbera hybrida*; At, *Arabidopsis thaliana*; Os, *Oryza sativa*; Ha, *Helianthus annuus*; Zm, *Zea mays*; Al, *Arabidopsis lyrata*. (**C**) Heatmap shows the relative expression levels of GhmiR156 at three developmental stages of ray florets (S1, S3 and S6). FPKM: fragments per kilobase of transcript per million mapped reads. Data are derived from three biological replicates. Expression levels are represented by a color scale (red, upregulation; blue, downregulation).

Through small RNA sequencing analysis of ray florets at three developmental stages in gerbera, 164 microRNA reads were identified. Comparative analysis with miRBase v22 revealed 24 known miRNAs and 140 novel miRNAs [46]. Based on these foundations and our laboratory’s research orientation, we focused on the putative function of GhmiR156 during the process of floral development. Firstly, we amplified its precursor sequence and predicted its secondary structure using RNAfold WebServer, and the analysis showed a stable hairpin structure with a minimum free energy (MFE) of −53.1 kcal/mol. Then, structural analysis found that pre-GhmiR156 forms a characteristic stem-loop configuration, with the mature GhmiR156 sequence positioned on the 5′ arm (Figure 1A).

Multiple sequence alignment of miR156 mature sequences across diverse plant species, including *Arabidopsis thaliana*, *Oryza sativa*, *Helianthus annuus*, *Zea mays* and *Arabidopsis lyrata*, revealed remarkable sequence conservation. DNAMAN software (version 7.0) analysis indicated 93.65% sequence similarity, with only a single base mismatch among the examined species (Figure 1B). This high degree of conservation suggests the evolutionary importance of miR156 in plant development. Moreover, expression profiling of GhmiR156 during different growth and development stages of ray florets showed an increased abundance (Figure 1C). The conserved nature of miR156, combined with its stage-specific expression profile, emphasizes its potential significance in gerbera floral development.

### 2.2. Identification of GhmiR156 Targets and Functional Characterization of GhSPL2

**Figure 2:**
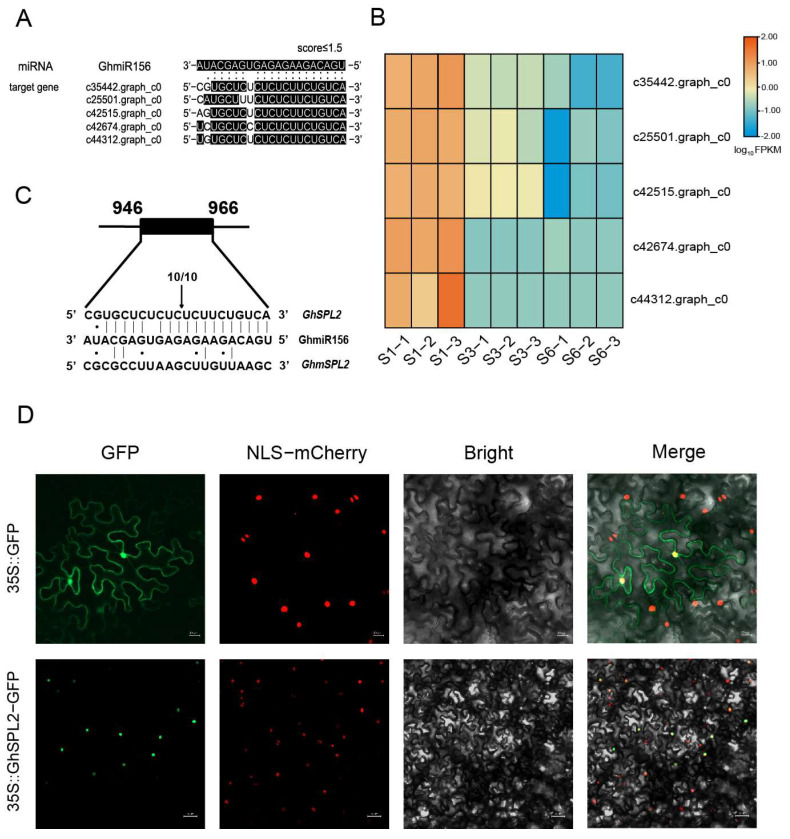
Prediction of the target relationship between *GhSPLs* and GhmiR156, identification and phylogenetic tree analysis of GhSPL2. (**A**) Predicted target sites of GhmiR156 in five genes. (**B**) Heatmap representation of the *GhSPLs* expression at three developmental stages of ray florets (S1, S3 and S6). FPKM: fragments per kilobase of transcript per million mapped reads. (**C**) Identification of the GhmiR156 target site with RLM-5′RACE. The cleaved site is marked with an arrow. The numbers above the arrow are the number of clones with an identified 5′ end detected in the total sequenced clones. (**D**) Subcellular localization of GhSPL2. GhSPL2 fused with GFP or GFP was transformed into tobacco leaves. Scale bar = 50 μm. Nuclei are represented by NLS-mCherry. Merged images show the co-localization of NLS and GFP signals.

miRNAs regulate gene expression primarily through transcript cleavage of their target genes [47]. In this study, target gene prediction identified 5 potential targets of GhmiR156 (score ≤ 1.5) (Figure 2A). Expression profiling revealed an inverse correlation between GhmiR156 abundance and its predicted targets, with target gene expression progressively declining during ray floret development (Figure 1C and Figure 2B). This correlation was further demonstrated by qRT-PCR analysis across six developmental stages of ray florets (stages 1–6) (Supplementary Figure S1).

To validate the miRNA-target interaction, we performed RNA ligase-mediated (RLM)-5′RACE. The assay confirmed that GhmiR156 mediated the cleavage of c35442.graph_c0 (gerbera genome accession: Gh_000076.m1), with the cleavage site precisely mapped between nucleotides 12 (C) and 13 (U) within the GhmiR156 complementary region (Figure 2C). In contrast, no cleavage events were detected for the other four potential targets. Based on the cleavage relationship, c35442.graph_c0 was selected for further investigation. Phylogenetic analysis revealed that this protein shares the highest sequence similarity with AtSPL2, and thus was named GhSPL2 (Supplementary Figure S2). Subsequently, expression profiles across different tissues and organs showed GhmiR156 and *GhSPL2* exhibited an opposite expression trend (Supplementary Figure S3). Furthermore, we also examined the subcellular localization of GhSPL2, revealing it to be a nuclear-localized protein (Figure 2D).

### 2.3. GhSPL2 Is a Direct Target of GhmiR156

**Figure 3:**
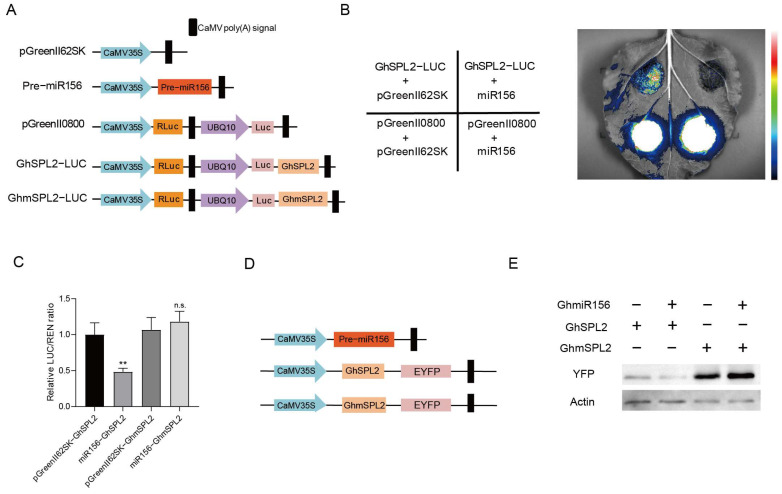
*GhSPL2* is a direct target of GhmiR156. (**A**) Schematic diagram of the dual-luciferase reporter assay vectors for GhmiR156 targeting *GhSPL2*. (**B**) *GhSPL2*-LUC was co-transformed with GhmiR156 in tobacco leaves. *GhSPL2*-LUC + miR156 was the experimental group, and the rest were the control group. Black: low fluorescence intensity; white: high fluorescence intensity. (**C**) GhmiR156 cleaves the *GhSPL2* mRNA by dual-luciferase reporter assay. Error bars represent the standard deviation of the three replicates (** *p* < 0.01; n.s., not significant). (**D**) Schematic diagram of the vector structure of the Western blotting assay. (**E**) Protein levels of GhSPL2 or GhmSPL2 during co-transforming with GhmiR156 by Western blotting.

To confirm the target relationship between GhmiR156 and *GhSPL2*, we conducted dual-luciferase assays to quantitatively assess this regulatory interaction. The 102 bp GhmiR156 precursor was cloned into the GhmiR156-62SK expression vector, while *GhSPL2*-LUC and *GhmSPL2*-LUC reporter constructs were also generated (Figure 3A). The result showed significant suppression of luciferase activity when *GhSPL2*-LUC was co-expressed with GhmiR156-62SK, compared to controls (Figure 3B). This suppression was abolished in the GhmSPL2-LUC construct, demonstrating the specificity of the interaction (Figure 3C). The results demonstrate that GhmiR156 directly targets *GhSPL2*.

Western blotting analysis of *GhSPL2*-YFP and *GhmSPL2*-YFP fusion proteins in Arabidopsis protoplasts provided additional experimental evidence. Co-expression with GhmiR156 significantly reduced *GhSPL2*-YFP protein levels, while *GhmSPL2*-YFP accumulation remained unaffected (Figure 3D,E). Collectively, these experiments demonstrate that GhmiR156 specifically targets *GhSPL2* through direct interaction at the predicted binding site, leading to transcript cleavage and subsequent protein degradation. The consistent results across multiple experimental systems provide compelling evidence for the target regulation of *GhSPL2* by GhmiR156 in gerbera.

### 2.4. Overexpression of GhSPL2 Inhibits Anthocyanin Accumulation in Ray Florets

**Figure 4:**
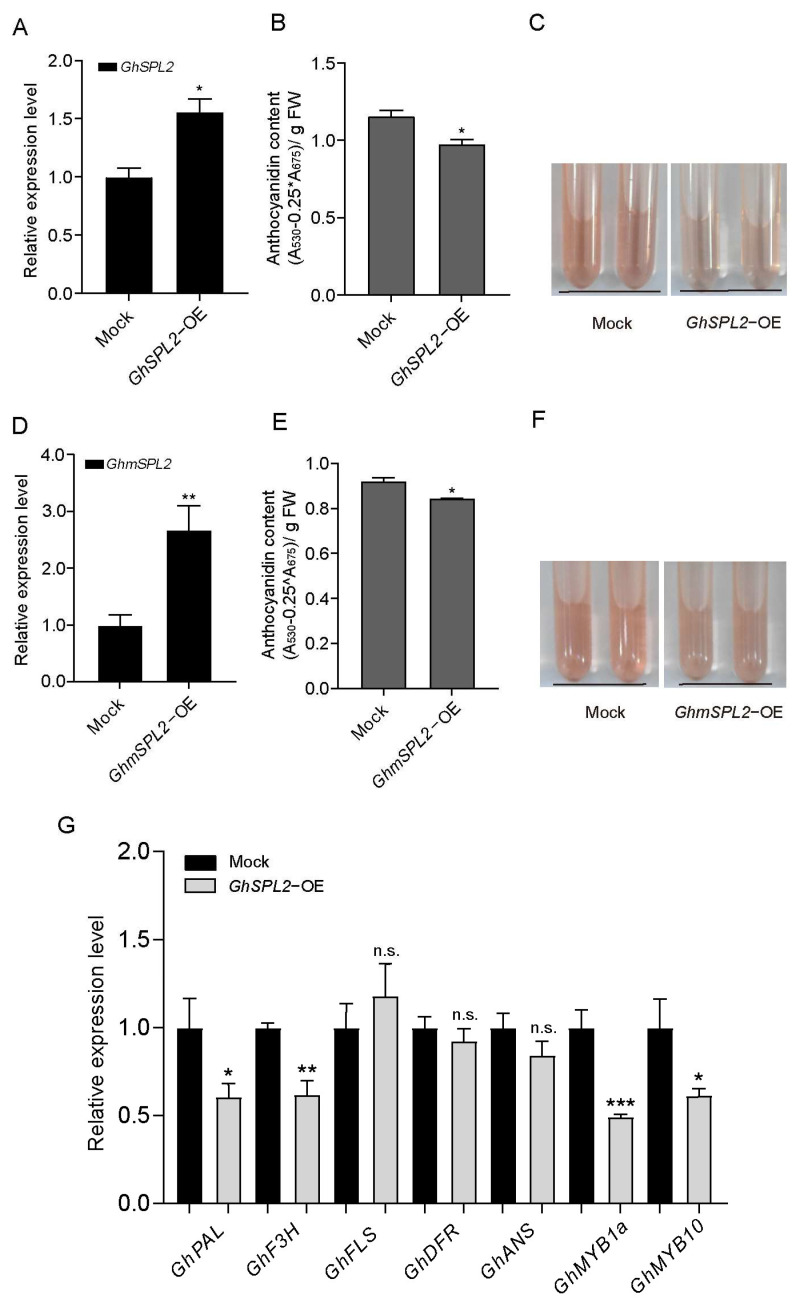
Overexpression of *GhSPL2* suppresses anthocyanin accumulation of ray florets. The relative expression levels of *GhSPL2* and *GhmSPL2* in the mock and *GhSPL2*-OE (**A**) and the mock and *GhmSPL2*-OE (**D**), respectively. The total anthocyanin content of ray florets in *GhSPL2*-OE (**B**) and *GhmSPL2*-OE (**E**). Anthocyanins extracted from *GhSPL2*-OE and the mock (**C**) and *GhmSPL2*-OE and the mock (**F**) by 1% (*v*/*v*) HCl-methanol, respectively. (**G**) The effects of *GhSPL2* overexpression on anthocyanin biosynthetic or regulatory genes were investigated using qRT-PCR analyses in stage 3 transient overexpression gerbera petals, with an empty vector used as the mock. Error bars represent the standard deviation of the three replicates (* *p* < 0.05, ** *p* < 0.01, *** *p* < 0.001; n.s., not significant).

Previous research has shown that *SPL*s play crucial roles in the juvenile-to-adult growth phase transition, flower morphogenesis and secondary metabolism regulation [32,48,49]. However, whether *GhSPL2* plays a similar role in gerbera remains unclear. To explore its function, we conducted transient overexpression of *GhSPL2* (*GhSPL2*-OE) and *GhmSPL2* (*GhmSPL2*-OE) in ray florets. qRT-PCR analysis showed successful overexpression of *GhSPL2* or *GhmSPL2* in transformed petals compared to mock (Figure 4A,D). Subsequent quantitative analysis of anthocyanin content revealed significantly lower anthocyanin accumulation in *GhSPL2*-OE petals compared to mocks (Figure 4B,C), providing crucial evidence that GhSPL2 functions as a negative regulator of anthocyanin biosynthesis in ray florets. Interestingly, overexpression of *GhmSPL2* also leads to a decrease in anthocyanin content (Figure 4E,F). This suggests that the protein encoded by the synonymous mutation of GhSPL2 retains its original function and still reduces anthocyanin accumulation in gerbera.

To elucidate the molecular mechanism underlying this regulatory role, we analyzed the expression levels of key anthocyanin biosynthetic genes in *GhSPL2*-OE petals. The result showed significant downregulation of several critical pathway genes, including *GhMYB10*, *GhMYB1a*, *GhPAL*, *GhF3H*, and *GhUFGT*. While *GhDFR* and *GhANS* expression also decreased, these changes were not statistically significant (Figure 4G). These findings demonstrate that *GhSPL2* may negatively regulate anthocyanin accumulation in gerbera ray florets through transcriptional repression of key biosynthetic genes.

### 2.5. GhmiR156 Relieves Negative Regulation by GhSPL2 in Ray Floret Anthocyanin Accumulation

**Figure 5:**
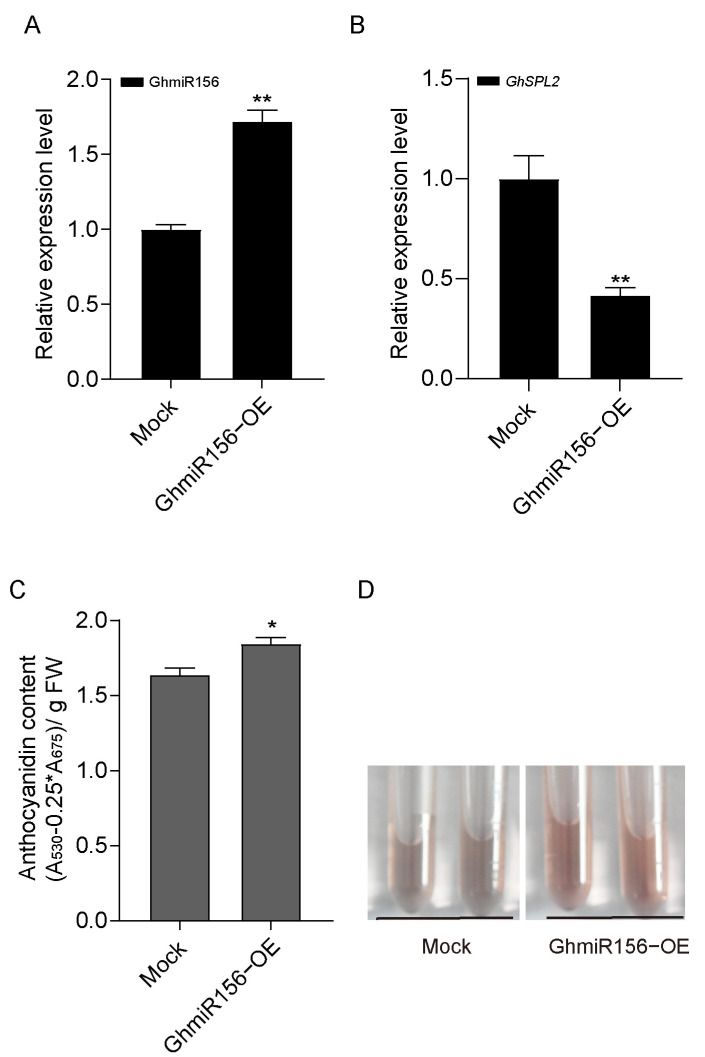
GhmiR156 relieves negative regulation by *GhSPL2* in ray floret anthocyanin accumulation. The relative expression levels of GhmiR156 (**A**) and *GhSPL2* (**B**) in GhmiR156-OE and the mock, respectively. (**C**) Total anthocyanin content in GhmiR156-OE and the mock. Error bars represent the standard deviation of the three replicates (* *p* < 0.05, ** *p* < 0.01; n.s., not significant). (**D**) Anthocyanins extracted from GhmiR156-OE and the mock by 1% (*v*/*v*) HCl-methanol.

To establish the functional relationship between GhmiR156 and *GhSPL2* in anthocyanin regulation, we conducted transient overexpression of GhmiR156 (GhmiR156-OE) in ray florets. Successful overexpression of GhmiR156 was confirmed by qRT-PCR (Figure 5A). Meanwhile, we found *GhSPL2* transcript levels decreased in GhmiR156-OE petals (Figure 5B).

The physiological consequence of this regulatory role was evident in anthocyanin accumulation patterns. The result showed higher anthocyanin content in GhmiR156-OE petals compared to mock (Figure 5C,D). This finding establishes a clear functional link between GhmiR156 expression and enhanced anthocyanin accumulation in gerbera ray florets, demonstrating that GhmiR156 serves as a positive regulator of anthocyanin biosynthesis through its negative regulation of *GhSPL2*, which in turn alleviates the suppression of anthocyanin biosynthetic genes.

## 3. Discussion

Accumulating evidence suggests that the miR156-*SPL*s pathway plays a significant role in the biosynthesis of anthocyanins [14,15,17,20,34,50]. However, the function of miR156-*SPLs* in petal coloration of the Asteraceae family is still poorly understood. In our previous analysis, we examined small RNA sequencing and transcriptome data, which allowed us to identify this pathway’s relevant miRNAs and their target genes involved in the growth and development of ray florets [46]. Our current study reveals that GhmiR156 directly targets *GhSPL2*, demonstrating its primary regulatory role in petal color by influencing the expression of several key genes involved in anthocyanin synthesis. Collectively, these findings underscore the crucial functions of the miR156-*GhSPL2* module in regulating anthocyanin biosynthesis in the ray florets of gerbera.

### 3.1. GhSPL2 Inhibits Anthocyanin Synthesis by Influencing the Expression of Anthocyanin-Related Genes

Previous studies have demonstrated that SPL transcription factors primarily suppress anthocyanin biosynthesis by downregulating the expression of key regulatory genes, such as *MYB*, *bHLH*, and *WD40*, which form the MYB-bHLH-WD40 (MBW) complex [13,17]. In this study, we observed that *GhMYB1a* and *GhMYB10* were significantly downregulated in *GhSPL2*-OE ray florets (Figure 4G), suggesting that *GhSPL2* may inhibit anthocyanin accumulation by repressing these MYB TFs. This finding aligns with reports in other plant species, where SPL TFs regulate MYB TFs to modulate anthocyanin biosynthesis. For instance, in litchi, *LcSPL1*, targeted by miR156, negatively regulates anthocyanin biosynthesis by interacting with LcMYB1 [51]. Similar regulatory mechanisms have been observed in red pear (*Pyrus* L.), grape (*Vitis vinifera* L.) and herbaceous peony [26,37,50], indicating that the function of *GhSPL2* in regulating MYB TFs may be conserved across species. In addition to GhMYB1a and GhMYB10, other MYB TFs, such as MYB9 and MYB114, have also been implicated in anthocyanin accumulation [16,52]. Therefore, further investigation is needed to determine whether additional MYB TFs are involved in *GhSPL2*-mediated inhibition of anthocyanin biosynthesis in gerbera ray florets.

Besides the downregulation of *GhMYB1a* and *GhMYB10*, the expression of key structural genes in the anthocyanin biosynthetic pathway, including *GhPAL*, *GhF3H*, and *GhUFGT*, was also downregulated in *GhSPL2*-OE ray florets (Figure 4G). This suggests that *GhSPL2* suppresses anthocyanin synthesis by directly or indirectly repressing the transcription of these structural genes. Previous studies have shown that the MBW complex or specific MYB TFs regulate anthocyanin biosynthesis by modulating the expression of structural genes [26,50,53]. Consequently, *GhSPL2* might directly bind to the promoters of structural genes in the early stage of the anthocyanin biosynthetic pathway or interfere with the activity of the MBW complex, thereby inhibiting anthocyanin biosynthesis [17,54]. Further studies are required to elucidate the precise molecular mechanisms by which *GhSPL2* regulates anthocyanin metabolism in gerbera ray florets.

### 3.2. GhmiR156 Targeting GhSPL2 Regulates Anthocyanin Biosynthesis in Gerbera

It is well known that various miRNAs regulate anthocyanin biosynthesis in various plant species, including miR858, miR828, miR156, miR399 and miR172 [17,19,55,56]. Among these, miR156 has been shown to play a significant role in fruit color changes in red pear, grape, and blueberry, as well as petal coloration in tree peony and lily [26,30,34,36,50]. However, the involvement of miR156 in anthocyanin biosynthesis of gerbera remains poorly understood. Building on our previous research, which identified differentially expressed miRNAs associated with the growth and development of ray petals [46], we investigated the expression pattern of GhmiR156 in this study (Supplementary Figure S1). We observed that the expression of GhmiR156 gradually increased during the developmental stages of ray florets, consistent with the predicted heatmap (Figure 1C). This suggests that GhmiR156 may play a critical role in the growth and development of ray florets.

The miR156-*SPL* module is known to play a pivotal role in regulating anthocyanin accumulation across various plant species [26,30,34]. For example, in Arabidopsis, miR156-regulated *SPL* suppresses anthocyanin accumulation [17,57,58]. Similarly, in blueberry, VcmiR156a targets *VcSPL12* to regulate fruit coloration [30], while in Chinese sand pear (*Pyrus* spp.), miR156 and its target *PpSPL* genes modulate light-induced red peel coloration and anthocyanin accumulation [15]. Research on the miR156-*SPL* module in plants of the Asteraceae family elucidates its molecular mechanism related to bicolor petal formation [59,60]. In this study, we predicted *GhSPL2* as a target of GhmiR156, and the RLM-5′RACE assay confirmed that GhmiR156 cleaves *GhSPL2* mRNA (Figure 2C). Further validation through dual-luciferase assay and Western blotting confirmed the regulatory relationship between GhmiR156 and *GhSPL2* (Figure 3). The overexpression of miR156 in GhmiR156-OE resulted in the degradation of *GhSPL2* and an increase in anthocyanin content (Figure 5). This effect contrasted sharply with the reduced anthocyanin content observed in *GhSPL2*-OE (Figure 4). These findings demonstrate that GhmiR156 represses *GhSPL2* expression, thereby alleviating its negative regulation on anthocyanin accumulation in gerbera ray florets. Furthermore, building upon previous functional research of transcription factors in anthocyanin biosynthesis in gerbera [59,60], our study provides the first mechanistic insight into how miR156 regulates this process, thereby contributing to a more refined molecular network connecting miRNAs, transcription factors and their target genes. Although studies in other plants have indicated SPLs might function redundantly [61,62,63], our study specifically focused on a subset of *SPLs* targeted by GhmiR156. Thus, it is plausible that other SPLs, beyond those regulated by miR156, contribute to flower color formation, which needs further investigation.

## 4. Materials and Methods

### 4.1. Plant Materials and Growth Conditions

In this study, *Gerbera hybrida* (*G. jamesonii* Bolus ex Adlam × *G. viridifolia* Schultz-Bip.) cultivars “Shenzhen No.5” and “Fenjiaren” were used. Seedlings were cultivated in a greenhouse with natural light (16 h light, 8 h dark), a temperature between 26 and 28 °C (day) and 18–20 °C (night), and relative humidity 65–80%. Ray florets at various developmental stages [38] were collected for experiments. Other plant samples were collected from various tissues at specific developmental stages. Root samples included old roots (brown, >15 cm in length) and young roots (white, newly formed, about 5 cm in length). Leaf samples comprised old leaves (fully expanded) and young leaves (newly grown, curly). For floral tissues, the receptacle, stem, and calyx were dissected from flowers at stage 2, while the ray florets, trans florets, and disk florets were collected from flowers at stage 5. Ray florets at stage 3 were used for transient transformation assays [43,45,64].

*Arabidopsis thaliana* (Columbia ecotype) seeds were surface-sterilized with 75% ethanol and 2% sodium hypochlorite, plated on Murashige and Skoog (MS) medium, and vernalized at 4 °C in the dark for 2 days. Plates were then transferred to a tissue culture room maintained at 22 ± 2 °C with a 16 h light/8 h dark cycle and 60–80% relative humidity. Seedlings were subsequently transplanted into soil and grown under the same conditions.

### 4.2. Bioinformatic Analysis

The precursor sequence of GhmiR156 was obtained from small RNA sequencing and transcriptome data [46], and its secondary structure was predicted using RNAfold WebServer (http://rna.tbi.univie.ac.at/cgi-bin/RNAWebSuite/RNAfold.cgi, accessed on 28 October 2021). Mature miR156 sequences from other species were downloaded from miRBase (https://www.mirbase.org/, accessed on 7 May 2023) and were aligned using DNAMAN 7.0. Target genes of GhmiR156 were predicted using psRNATarget (https://www.zhaolab.org/psRNATarget/, accessed on 4 September 2021) with gerbera transcriptome data. FPKM (fragments per kilobase of transcript per million mapped reads) and differential gene expression analyses were carried out following previously reported methods [65]. Heatmaps were generated using TBtools software (version 2.210).

### 4.3. Cloning and Bioinformatics Analysis of GhSPL2

The full-length coding sequence of *GhSPL2* was amplified from gerbera cDNA using gene-specific primers (*GhSPL2*-F1/R1; Supplementary Table S1). Conserved domains were predicted using the NCBI database. Amino acid sequence alignment with SPL homologs from other plant species was performed using DNAMAN 7.0, and phylogenetic analysis was conducted using MEGA 7.0. A neighbor-joining phylogenetic tree was constructed with 1000 bootstrap replicates.

### 4.4. RNA Extraction and Quantitative RT-PCR (qRT-PCR)

Total RNA was extracted from ray florets and other tissues using Trizol reagent (Invitrogen (Carlsbad, CA, USA), Cat. No. 15596-026). First-strand cDNA was synthesized using the ReverTra Ace qPCR RT Kit (TOYOBO (Osaka, Japan), Cat. No. FSQ-301), following the manufacturer’s procedure. For miRNA quantification, the Mir-X miRNA First-Strand Synthesis Kit (Clontech (San Jose, CA, USA), Cat. No. 638315) was used. Quantitative real-time PCR (qRT-PCR) was performed on a CFX96 Touch^TM^ Real-Time PCR Detection System (Bio-Rad Laboratories, Hercules, CA, USA) using 2× RealStar Green Fast Mixture (GenStar (San Francisco, CA, USA), Cat. No. A301-01). Primer pairs are listed in Supplementary Table S1. *GhACTIN* (AJ763915) and small nuclear RNA (snRNA) U6 served as internal controls for normalization [66]. Data were analyzed using the 2^−∆∆Ct^ method [67], with three biological and technical replicates per sample.

### 4.5. Subcellular Localization of GhSPL2

The full coding sequence of *GhSPL2* was cloned into the pCAMBIA 1301-GFP vector to generate the 35S::*GhSPL2*-GFP. This construct and the empty pCAMBIA 1301-GFP vector were separately introduced into tobacco (*Nicotiana benthamiana*) leaves expressing NLS-mCherry. Fluorescence signals were observed using a confocal laser-scanning microscope (LSM 710, Zeiss, Oberkochen, Germany) after 2 days of incubation.

### 4.6. Dual-Luciferase Reporter Assay

The dual-luciferase assay was performed as previously described [68,69]. The open reading frame (ORF) of *GhSPL2* was cloned into the pGreenII 0800-LUC vector to generate *GhSPL2*-LUC. A synonymous mutation in *GhSPL2* was introduced by PCR to create *GhmSPL2* (GhmiR156 targeting sites in *GhSPL2* were replaced with synonymous mutation bases, as shown in Figure 2C. These modifications preserved the amino acid sequence while disrupting the miRNA-target interaction, as confirmed by the inability of psRNATarget to predict the interaction post-mutation, which was also cloned into pGreenII 0800-LUC. The GhmiR156 precursor was inserted into pGreenII 62SK to produce GhmiR156-62SK. All constructs were co-transformed into tobacco leaves via *Agrobacterium*-mediated transformation. These plasmids were also co-transformed into Arabidopsis leaf protoplasts. Three biological replicates were performed for each experiment.

### 4.7. Western Blotting Analysis

To assess whether GhmiR156 inhibits GhSPL2 protein accumulation, the full-length coding sequences of *GhSPL2* and *GhmSPL2* were cloned into the pSAT6-YFP vector at *Eco*I/*Kpn*I sites to generate *GhSPL2*-YFP and *GhmSPL2*-YFP constructs. These constructs were co-transformed with GhmiR156 into Arabidopsis leaf protoplasts, with *GhSPL2*-YFP or *GhmSPL2*-YFP alone serving as controls. Proteins were denatured, separated on a 7.5% SDS-PAGE gel, and transferred to a PVDF membrane. After blocking, anti-YFP antibodies (1:10,000 dilution) and goat anti-mouse secondary antibodies were used for detection. Immunoblots were visualized using chemiluminescence.

### 4.8. RNA Ligase-Mediated 5′ RACE(RLM-5′RACE)

RLM-5′RACE was conducted as previously described [46]. Total RNA from ray florets was ligated to a 5′ adapter sequence using T4 RNA ligase (Takara, Cat. No. 2050A). cDNA was synthesized using the ReverTra Ace qPCR RT Master Mix with gDNA Remover Kit (Toyobo, Cat. No. FSQ-301). PCR products were cloned into the pMD18-T vector (TaKaRa, Dalian, China), and 15 positive clones were sequenced.

### 4.9. Transient Transformation of Ray Florets

Transient transformation was performed as described [43,45]. The full coding sequences of *GhSPL2* and *GhmSPL2* were cloned into pCAMBIA1301 to generate overexpression vectors. The GhmiR156 precursor was also cloned into pCAMBIA1301. These constructs were introduced into the *Agrobacterium tumefaciens* strain C58C1. Cultures were grown in LB medium with 20 μM acetosyringone (AS) and resuspended in infiltration buffer (10 mM MES, 200 μM AS, 10 mM MgCl_2_, pH 5.6). Ray florets were vacuum-infiltrated and cultured under controlled conditions. Each treatment contained three biological replicates, and each replicate consisted of a pooled sample from five flowers, including approximately 80 petals.

### 4.10. Measurement of Total Anthocyanin Content

Anthocyanin content was measured as described [70]. Ray florets were weighed and extracted in a methanol-hydrochloric acid solution (99:1, *v*/*v*) at 4 °C for 48 h. Absorbance was measured at 530 nm (A_530_) and 657 nm (A_657_), and total anthocyanin content was calculated using the formula (A_530_ − 0.25 × A_657_)/g fresh weight.

### 4.11. Statistical Analysis

qRT-PCR data, LUC/REN ratio and anthocyanin content are presented using bar graphs. Data were analyzed using Student’s *t*-test in GraphPad Prism 9. Error bars represent standard deviation (SD). Significance levels are indicated as * *p* < 0.05, ** *p* < 0.01, and *** *p* < 0.001. All figures were assembled using Adobe Illustrator 2016 (Adobe Inc., San Jose, CA, USA).

## 5. Conclusions

In this study, we verified a molecular module, GhmiR156-*GhSPL2*, linked to anthocyanin biosynthesis from gerbera. Multiple results confirmed the regulatory relationship between GhmiR156 and *GhSPL2*. The overexpression of *GhSPL2* in gerbera ray petals resulted in a decrease in anthocyanin content. However, the overexpression of GhmiR156 resulted in the degradation of *GhSPL2* and an increase in anthocyanin content. These findings suggest that GhmiR156-*GhSPL2* plays a pivotal role in petal coloration and lays the foundation for research on regulating flower color of ornamental plants.

## Data Availability

The original contributions presented in this study are included in the article/Supplementary Material. Further inquiries can be directed to the corresponding author.

## Supplementary Materials

The following supporting information can be downloaded at: https://www.mdpi.com/article/10.3390/ijms27010318/s1.
