# Supplementary material for: GhmiR156-GhSPL2 Module Regulates Anthocyanin Biosynthesis of Ray Florets in Gerbera hybrida
Source: Int J Mol Sci. 2025 Dec 27;27(1):318. doi: 10.3390/ijms27010318 (PMC12786271; doi:10.3390/ijms27010318)
Supplement: Supplementary file 1 [file ijms-27-00318-s001.zip › Supplementary files/supplementary file 2.pdf]

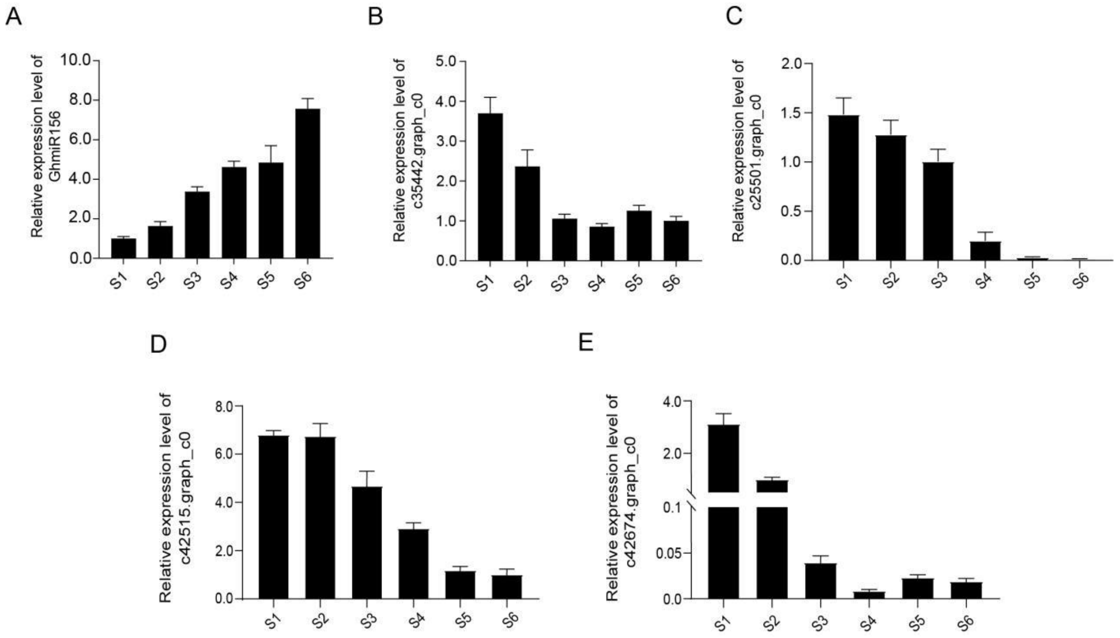

Supplementary figure 1 The relative expression patterns of GhmiR156 (A), c35442.graph_c0 (B), c25501.graph_c0 (C), c42515.graph c0 (D) and c42674.graph c0 (E) in the petals of gerbera during different developmental stages (S1 to S6). The expression level of c44312.graph c0 was undetectable due to its low abundance across six floral development stages.

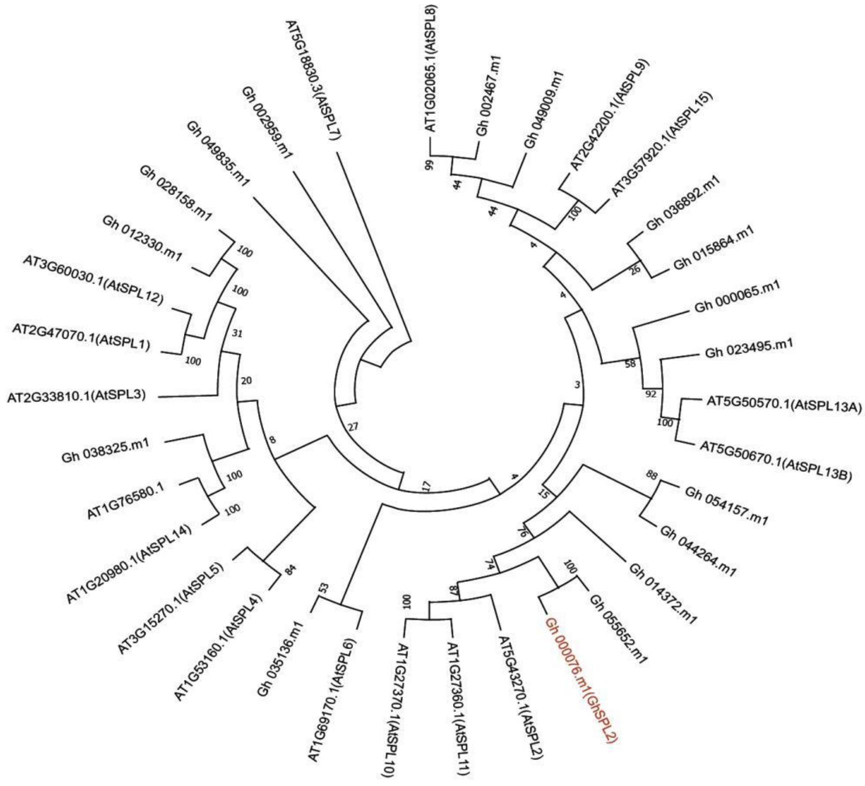

Supplementary figure 2 Phylogenetic tree containing 17 GhSPLs and AtSPLs constructed using the neighbor-joining method with 1000 bootstrap replications.

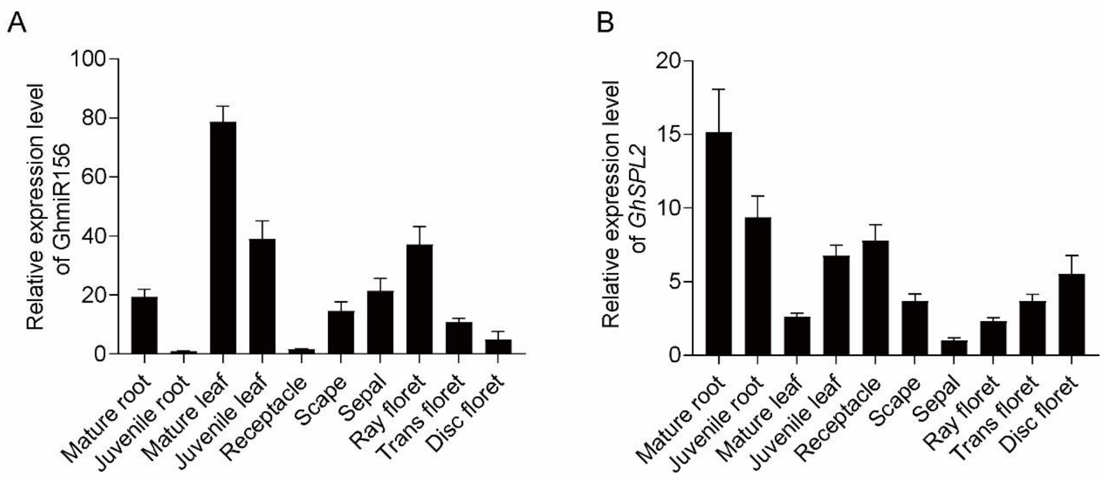

Supplementary figure 3 The expression patterns of GhmiR156 and *GhSPL2*. Tissue specific expression of GhmiR156 (A) and *GhSPL2* (B) in gerbera. Root samples included old roots (brown, >15 cm in length) and young roots (white, newly formed, about 5 cm in length). Leaf samples comprised old leaves (fully expanded) and young leaves (newly grown, curly). For floral tissues, the receptacle, stem, and calyx were dissected from flowers at stage 2, while the ray florets, trans florets, and disc florets were collected from flowers at stage 5.
